# Worldwide ophthalmological research production 2000–2020, with special focus on the Nordic contribution

**DOI:** 10.1111/aos.15200

**Published:** 2022-06-07

**Authors:** Tomas Bro

**Affiliations:** ^1^ Department of Biomedical and Clinical Sciences Linköping University Linköping

**Keywords:** bibliometrics, journal impact factor, ophthalmology, publication productivity

## Abstract

**Purpose:**

To explore the trends in worldwide ophthalmic research production over a 21‐year period in relation to journals, contributing countries and dominating topics with special focus on the Nordic region.

**Methods:**

Articles published between 2000 and 2020 in 20 top‐ranked ophthalmology journals were included. Number of articles and impact points were measured per country for each year. The most frequently occurring keywords were calculated worldwide and for the top five contributing countries and the Nordic countries. Trends were explored using linear regression.

**Results:**

The analysis included 65 220 articles. Linear regression showed an increase with 56 articles per year (β = 56.3, *R* ^2^ = 0.72, p‐value < 0.01). The United States published the most articles, comprising 35% of the worldwide total, followed by the United Kingdom (9%) and Japan (7%). Population‐adjusted productivity revealed that Iceland was the most prolific country with 10 articles per million inhabitants/year. Singapore was second and Denmark third with corresponding numbers of nine and seven. Analysing regional trends, Asia had the largest increase in yearly number of articles (β = 29.1, *R* ^2^ = 0.89, p‐value < 0.01). The strongest positive trend was observed in China (β = 15.7, *R* ^2^ = 0.94, p‐value < 0.01). The Nordic countries contributed with 3.6% of worldwide ophthalmological papers. Among these, Denmark was the only country with a significant positive trend in impact points per million inhabitants per year (β = 0.6, *R* ^2^ = 0.54, p‐value < 0.01). The most frequently occurring eye disease within the whole time frame was myopia (5.8%) followed by macular degeneration (5.4%) and glaucoma (5.3%). Linear regression showed a significant increase in the proportion of articles about diabetic retinopathy (β = 0.2%, *R* ^2^ = 0.88, p‐value < 0.01) a significant decrease in the proportion in articles about cataract (β = −0.1%, *R* ^2^ = 0.70, p‐value < 0.01) and myopia (β = −0.1%, *R* ^2^ = 0.67, p‐value < 0.01).

**Conclusions:**

The worldwide ophthalmic research productivity has maintained a growing trend from 2000 to 2020. While North America and Europe are the major contributors, the scientific activity in Asia and especially China is growing impressively. With the current progress, Asia is forecast to outweigh Europe in 2025 and North America in 2033. Diabetic retinopathy was the most common eye disease in ophthalmologic papers in 2020, and also the topic with the strongest positive trend during 2000–2020.

## Introduction

In modern science, researchers from all fields are obliged to demonstrate their work to funding agencies and academic institutions. However, assessment of scientific quality is a notoriously difficult undertaking. With limited resources and increased bureaucratization of science, evaluation by peer review is therefore increasingly getting replaced by bibliometric measures (Haustein & Larivière 2015). One such metric is total impact factor points, which measures a researcher's published articles multiplied by the journal's impact factor at the time of publication. Journal impact factor in turn is defined as the average number of times papers published in the journal in the previous 2 years have been cited in the current year (Garfield 1972). Originally developed to select the most relevant journals to include in a citation database, it is today used extensively by leading journals in their advertising. Questions have been raised as to whether the impact factor represents an accurate measure of a journal's scientific quality and also whether the measure could be subject to inflation, mainly caused by an increased number of mean references. Nevertheless, increasing the impact factor is a fundamental editorial strategy for many journals (Neff & Olden 2010).

Previously published bibliographical research within the field of ophthalmology includes studies of specific journals (Chou *et al*. 2009), certain diseases (Caglar *et al*. 2016), the contribution of individual countries (Kumaragurupari *et al*. 2010), or other larger regions (Ugolini *et al*. 2004). A consistent finding of these papers is an increase in the yearly number of ophthalmologic papers during the past decades. The same trend has also been demonstrated in studies of the global research field of ophthalmology (Guerin *et al*. 2009; Yu *et al*. 2017) with an especially increased contribution from China (Huang *et al*. 2013; Schulz *et al*. 2016). During the same time, the mean impact factor for ophthalmological journals has also been increasing (Mansour *et al*. 2015). Studies of keywords have found that glaucoma, diabetic retinopathy and macular degeneration are diseases that attract the largest number of articles within the field (Kumar *et al*. 2011; Boudry *et al*. 2016).

However, no study has yet studied the global bibliometric trends in ophthalmology for the past 5 years. Comparisons of topics of ophthalmologic research interest have further never before been analysed between different countries, nor have trends for the factors that determine impact factor. The aim of this study was to perform an analysis of the worldwide research productivity within the field of ophthalmology during the past two decades, with an analysis of popular topics by country, and with special focus on the contribution from the Nordic countries.

## Method

Initially, the 20 highest ranked ophthalmological journals by Scimago Journal & Country rank (SCImago 2021) year 2020 were identified. Thereafter, full record data for all published articles in these journals from 2000 to 2020 was exported from the Web of Science core collection. If a journal changed name during the time frame, both names were used. Only research articles and reviews with a Pubmed ID were included. Letters to the editor, editorials, historical articles, bibliographies, meeting abstracts, book chapters, news and notes were excluded. The R package Bibliometrix was used to convert the export files data into dataframes and to identify the country of affiliation for the first author. The impact factor for each year was retrieved from Clarivate Journal Citation Reports (Clarivate Analytics 2021). If information was lacking a certain year, this was calculated manually using data from Scopus Citation Overview (Scopus 2022). As impact factor might be subject to inflation (Neff & Olden 2010), impact points were calculated by multiplying the number of articles with the mean impact factor for the whole time frame. Otherwise, newer articles would have a higher weight than older. The data were stratified into both journals and country of affiliation of the first author. To adjust research output to the size of a nation, the number of articles and impact points were divided by the average population size for the whole time frame using data from Gapminder. In trend analysis, the population size for each year was used (Gapminder 2021).

To analyse trends in topics, an analysis of MeSH terms was performed. MeSH stands for Medical Subject Headings and is a hierarchically organized vocabulary developed by The United States National Library of Medicine. The MeSH terms located beneath ‘eye disease’ were of certain interest and the occurrences of these were counted to compare trends in research areas both between countries and over time. The terms were retrieved from Pubmed using the R package EasyPubMed and MeshR within the Bioconductor package.

Linear regression analysis was conducted to explore worldwide trends in the number of articles and impact points from different regions, authors per article, references per article, impact factor for the included journals and for the proportion of the most frequent eye disease MeSH terms. The weighted mean impact factor within the dataset were decomposed using previous described methods (Althouse *et al*. 2009).

## Result

**Fig. 1:**
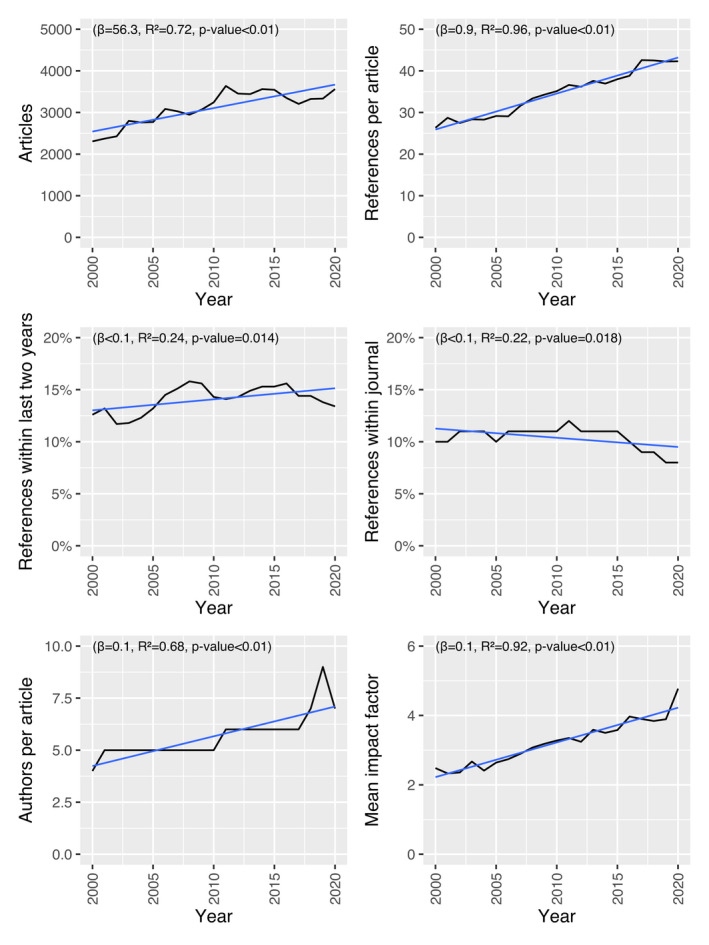
World trends for articles, number of authors, references, and impact factor for 20 top‐ranked ophthalmological journals 2000–2020 with linear regression. [Colour figure can be viewed at wileyonlinelibrary.com]

During 2000–2020, the total number of articles included for analysis published in the 20 chosen journals was 65 220. Information about impact factor was found in Clarivate Journal Citation Reports, except for *International Journal of Retina and Vitreous* 2019–2020 and *Ophthalmology Retina* 2019–2020 whereupon this was calculated manually with data from Scopus. The journals' mean impact factor during the time frame ranged from 1.29 to 9.83. *Investigative Ophthalmology and Visual Science* was the largest journal, contributing to more than 23% of all articles, and 26% of all ophthalmologic impact points. Mean number of authors ranged from 2.1 to 8.0 and mean number of references from 23 to 225. Journals mainly publishing review articles (*Progress in Retinal and Eye Research*, *Annual Review of Vision, Science Survey of Ophthalmology* had all mean number of references over 100 (Table 1). Regression analysis revealed significant positive trends for worldwide number of articles (β = 56.3, *R* ^2^ = 0.72), mean authors per article (β = 0.1, *R* ^2^ = 0.68), mean references per article (β = 0.9, *R* ^2^ = 0.96) and weighted mean impact factor (β = 0.1, *R* ^2^ = 0.92) (Fig. 1). Using methods described by Althouse et al., 1.7% of the mean yearly increase in impact factor of 4.8% could be explained by changes within the dataset. Changes in the number of articles contributed with 0.4%, citations 2.5%, proportion of citations within 2 years 0.9% and proportion of citations within 2 years to the dataset with −2.1% (as this measure decreased during the time frame).

**Table 1:** Data for articles and impact for 20 top‐ranked ophthalmologic journals 2000–2020.

Rank	Journal (first year later than 2000)	Total articles published	Mean impact factor	Total impact points	Mean authors	Mean references
1	*Progress in Retinal and Eye Research*	663	9.82	6511	4.2	225
2	*Ocular Surface (2005)*	539	6.38	3439	6.1	79
3	*Ophthalmology*	4941	5.58	27 571	7.3	32
4	*Annual Review of Vision Science (2015)*	129	5.2	671	2.1	140
5	*JAMA Ophthalmology*	3960	3.82	15 127	8.0	26
6	*Investigative Ophthalmology & Visual Science*	15 102	3.66	55 273	6.3	43
7	*Survey of Ophthalmology*	1043	3.43	3577	3.8	104
8	*American Journal of Ophthalmology*	6678	3.30	22 037	5.8	24
9	*Retina*	4525	2.89	13 077	5.6	26
10	*British Journal of Ophthalmology*	6448	2.84	18 312	5.8	25
11	*Current Opinion in Ophthalmology (2001)*	1243	2.78	3456	2.4	47
12	*Translational Vision Science & Technology (2012)*	1100	2.7	2970	6.9	38
13	*Journal of Refractive Surgery*	2104	2.59	5449	4.4	23
14	*Acta Ophthalmologica*	3590	2.48	8903	5.2	32
15	*Journal of Cataract & Refractive Surgery*	5359	2.47	13 237	4.4	23
16	*Eye*	3735	2.12	7918	4.9	30
17	*International Journal of Retina and Vitreous (2019)*	107	2.00	214	5.9	32
18	*Journal of Clinical and Experimental Ophthalmology*	2096	1.92	4024	4.7	30
19	*Ophthalmology Retina (2017)*	462	1.54	711	7.4	29
20	*Ophthalmologica*	1396	1.29	1801	4.9	29
	**Worldwide**	**65 220**	**3.29**	**206 569**	**6.0**	**35**

Worldwide numbers are bolded.

The US published the most articles, comprising 35% of the worldwide total, followed by the United Kingdom (9%) and Japan (7%). The five most productive countries provided almost two‐thirds (62%) of global publications. Population‐adjusted productivity revealed that Iceland was the most prolific country with 10 articles per million inhabitants and year. Singapore was second and Denmark third with corresponding numbers of nine and seven. Calculating impact points per million inhabitants, the top three countries were Singapore (31), Iceland (31) and Australia (20). Together the Nordic countries contributed with 3.6% of all ophthalmological articles and 3.0% of ophthalmological impact points (Table 2).

**Table 2:** Top 20 countries of scientific contribution to ophthalmological literature by articles and impact points in relation to worldwide research output and per inhabitants.

Rank	Country (percentage of worldwide articles)	Country (percentage of worldwide impact points)	Country (articles/million inhabitants/year)	Country (impact points/million inhabitants/year)
1	United States (35.2%)	United States (39.2%)	**Iceland (10)**	Singapore (31)
2	United Kingdom (8.9%)	United Kingdom (8.2%)	Singapore (9)	**Iceland (31)**
3	Japan (7.2%)	Japan (7.2%)	**Denmark (7)**	Australia (20)
4	China (6.0%)	China (5.6%)	Australia (6)	**Denmark (18)**
5	Germany (4.7%)	Germany (4.4%)	Austria (5)	Ireland (15)
6	Australia (4.5%)	Australia (4.4%)	Israel (5)	Austria (14)
7	South Korea (3.2%)	South Korea (3%)	Ireland (5)	Israel (14)
8	Italy (2.8%)	Italy (2.5%)	New Zealand (5)	United States (13)
9	Spain (2.5%)	Spain (2.3%)	United States (4)	United Kingdom (13)
10	India (2.4%)	France (2.2%)	United Kingdom (4)	Switzerland (13)
11	France (2.2%)	India (2.1%)	Netherlands (4)	New Zealand (13)
12	Canada (1.9%)	Canada (2%)	**Sweden (4)**	Netherlands (12)
13	Netherlands (1.9%)	Netherlands (2%)	Switzerland (4)	**Sweden (11)**
14	Singapore (1.5%)	Singapore (1.6%)	**Finland (3)**	**Finland (10)**
15	Turkey (1.5%)	Austria (1.2%)	Japan (2)	Japan (6)
16	**Sweden (1.3%)**	Turkey (1.1%)	Germany (2)	South Korea (6)
17	Austria (1.3%)	**Sweden (1.1%)**	South Korea (2)	Canada (6)
18	**Denmark (1.2%)**	**Denmark (1.0%)**	Spain (2)	**Norway (6)**
19	Switzerland (1.1%)	Switzerland (1.0%)	Canada (2)	Germany (5)
20	Israel (1.1%)	Israel (1.0%)	Greece (2)	Spain (5)

The Nordic countries are bolded.

**Fig. 2:**
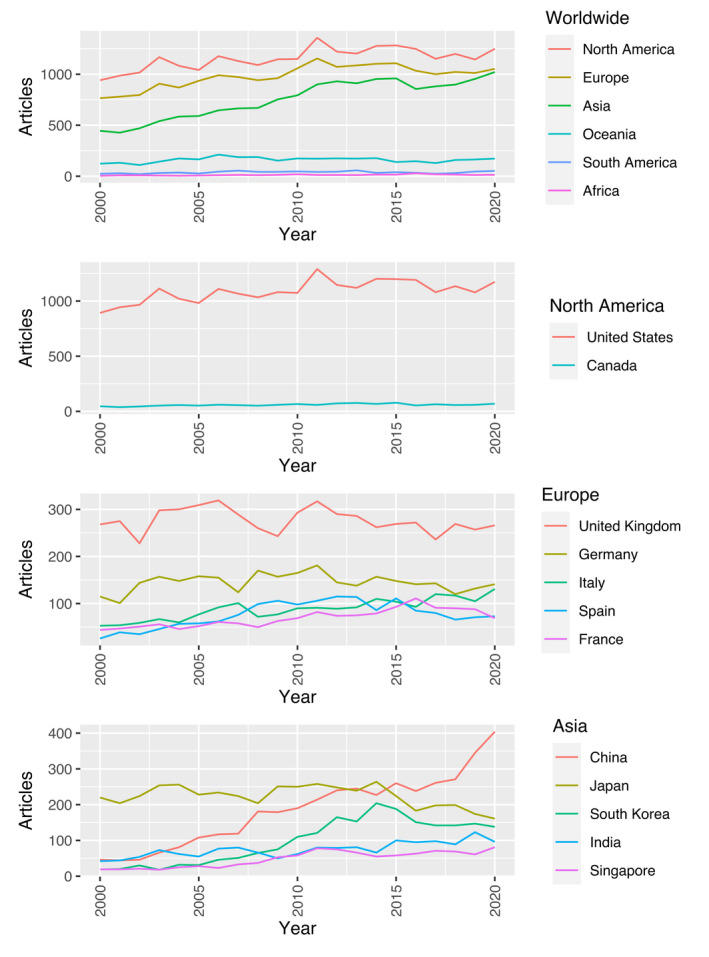
Trends in number of ophthalmological articles in worldwide regions and from top countries in the three most productive regions. [Colour figure can be viewed at wileyonlinelibrary.com]

**Fig. 3:**
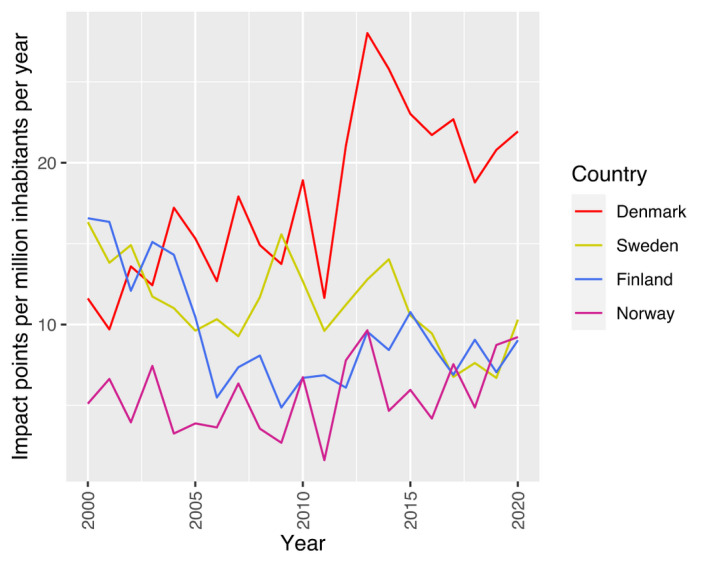
Trends in ophthalmological impact factor points for the Nordic countries 2000–2020. (Iceland is intentionally excluded due to high variance of this measure caused by a small population. Impact points were calculated for the given journal's mean impact factor for the whole time frame.) [Colour figure can be viewed at wileyonlinelibrary.com]

Analysing global regions, Asia had the largest increase in yearly number of articles (β = 29.1, *R* ^2^ = 0.89, p‐value < 0.01), followed by Europe (β = 13.7, *R* ^2^ = 0.58, p‐value < 0.01) and North America (β =11.9, *R* ^2^ = 0.48, p‐value  < 0.01). North America was dominated by the United States, producing 95% of all articles from the region. In Europe, significant positive trends with regression analysis were shown for Italy (β = 3.3, *R* ^2^ = 0.81, p‐value = <0.01) and France (β = 2.5, *R* ^2^ = 0.70, p‐value < 0.01). The trends in the remaining European countries could not be fitted into a linear regression model. In Asia, China had the strongest positive trend (β = 15.7, *R* ^2^ = 0.94, p‐value = <0.01) followed by South Korea (β = 8.8, *R* ^2^ = 0.76, p‐value < 0.01), Singapore (β = 3.2, *R* ^2^ = 0.78, p‐value < 0.01) and India (β = 2.8, *R* ^2^ = 0.68, p‐value < 0.01). Japan demonstrated a declining trend that could not be fitted to a linear regression model (Fig. 2). Among the Nordic countries, Denmark was the only country with a significant positive trend in impact points per million inhabitants per year (β = 0.6, *R* ^2^ = 0.54, p‐value < 0.01) with an especially strong increase between 2012 and 2014 (Fig. 3). Analysing articles, Denmark produced 57% more articles per year 2011–2021 (47) compared with 2000–2010 (30).

**Fig. 4:**
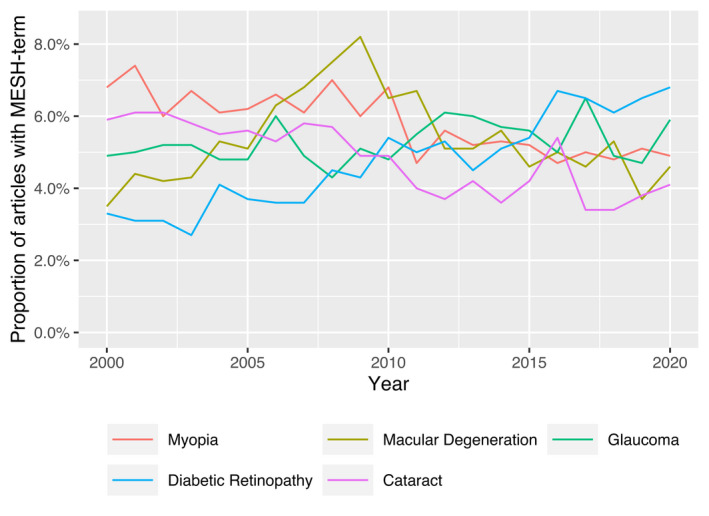
Trends in global occurrence of the five most common ‘eye disease’ MeSH terms within the ophthalmological literature. [Colour figure can be viewed at wileyonlinelibrary.com]

Of all included articles, 98% were classified with MeSH‐codes. Out of these, 81% had at least one term within the MeSH‐tree eye diseases. The most popular term was myopia, occurring in 5.8% of all MeSH‐termed articles, followed by macular degeneration (5.4%) and glaucoma (5.3%) (Table 3). Myopia also dominated the ophthalmologic research in China, occurring in twice as many articles (12.7%) as the second most frequent eye disease, cataract (6.0%). In Sweden, myopia was instead the 19th most frequent eye term, and cataract the most popular (11.9%), nearly twice as frequent as the second specified eye disease, glaucoma (6.4%). In Denmark, diabetic retinopathy was twice as frequent (14.2%) as the second specified eye disease of macular degeneration (6.8%) (Table 3). Linear regression of the five most frequent MeSH terms showed a significant increase in the proportion of articles about diabetic retinopathy (β = 0.2%, *R* ^2^ = 0.88, p‐value < 0.01) a significant decrease in the proportion in articles about cataract (β = −0.1%, *R* ^2^ = 0.70, p‐value < 0.01) and myopia (β = −0.1%, *R* ^2^ = 0.67, p‐value < 0.01). Remaining terms could not be fitted to a linear model (Fig. 4).

**Table 3:** The five most popular MeSH terms in the tree ‘eye diseases’ in ophthalmological publications 2000–2021 worldwide, from the five most productive countries and the Nordic countries.

	Rank				
	1	2	3	4	5
Worldwide (63933)	Myopia (5.8%)	Macular Degeneration (5.4%)	Glaucoma (5.3%)	Diabetic Retinopathy (4.8%)	Vision Disorders (4.8%)
United States (22272)	Glaucoma (6.2%)	Macular Degeneration (6.2%)	Vision Disorders (5.4%)	Retinal Diseases (4.9%)	Glaucoma, Open‐Angle (4.5%)
United Kingdom (5738)	Glaucoma (7.1%)	Vision Disorders (6.4%)	Cataract (5.8%)	Macular Degeneration (5.7%)	Diabetic Retinopathy (4.5%)
Japan (4629)	Diabetic Retinopathy (6.2%)	Retinal Perforations (5.7%)	Macular Oedema (5.7%)	Retinal Detachment (5.7%)	Retinal Diseases (5.2%)
China (3822)	Myopia (12.7%)	Cataract (6.0%)	Diabetic Retinopathy (5.4%)	Retinal Detachment (4.7%)	Myopia, Degenerative (4.2%)
Germany (2990)	Macular Degeneration (8.5%)	Myopia (5.8%)	Retinal Diseases (5.1%)	Glaucoma, Open‐Angle (4.7%)	Cataract (4.3%)
Denmark (774)	Diabetic Retinopathy (14.2%)	Macular Degeneration (6.8%)	Macular Oedema (6.7%)	Myopia (6.2%)	Wet Macular Degeneration (4.7%)
Sweden (826)	Cataract (11.9%)	Vision Disorders (9.4%)	Glaucoma, Open‐Angle (6.4%)	Glaucoma (5.8%)	Retinopathy of Prematurity (4.6%)
Finland (373)	Myopia (8.8%)	Glaucoma, Open‐Angle (8%)	Cataract (6.7%)	Exfoliation Syndrome (6.7%)	Diabetic Retinopathy (6.2%)
Norway (234)	Myopia (7.3%)	Cataract (6.0%)	Exfoliation Syndrome (5.1%)	Diabetic Retinopathy (4.7%)	Glaucoma, Open‐Angle (4.7%)
Iceland (67)	Diabetic Retinopathy (16.4%)	Macular Degeneration (13.4%)	Exfoliation Syndrome (10.4%)	Retinal Vein Occlusion (10.4%)	Blindness (7.5%)

## Discussion

The worldwide ophthalmic research productivity has maintained a growing trend from 2000 to 2020. While North America and Europe are major contributors, the scientific activity in Asia and especially China is growing impressively. With the current progress, Asia is forecast to outweigh Europe in 2025 and North America in 2033. Diabetic retinopathy was the most frequently occurring eye disease in ophthalmologic papers in 2020, and also the topic with the strongest positive trend during the time frame.

As in other fields, an increase in mean authors was seen, hopefully reflecting scientific progress and advances in study design more than changes in authorship criteria (Fontanarosa *et al*. 2017). Both the increase of studies and the increased contribution from China is in line with previous studies with shorter time periods (Guerin *et al*. 2009; Huang *et al*. 2013; Schulz *et al*. 2016; Yu *et al*. 2017). China's high production of science is further not limited to life science but has also been demonstrated in, for example, chemistry and physical science (Nature‐Index. 2021). In 2019, China also ranked as high or higher than the United States, in the top 1% of the world's most‐cited articles, after passing the EU in this measure in 2015. China has increased the research expenditures from 1.9% to 2.4% of gross domestic product during 2000–2020 (OECD 2022) and has a fast growth of clinical research centres (Wu *et al*. 2018). This science policy has propelled the country to operate at world‐leading levels of scientific output both in volume and quality, in a very short time period (Wagner *et al*. 2022). Japan, on the other hand, showed a decrease in scientific activity, with 38% fewer articles produced in 2016–2020 compared with 2010–2015. The number of scientific articles from Japan has also decreased dramatically in all fields during the 21st century. Decreasing birth rate, ageing population, economic stagnation and no increase in funding for research and development are possible causes for this decline (Shimokawa *et al*. 2017). While the United Kingdom and Germany had a relatively stable production of articles, Italy and France increased their scientific output. A likely explanation for this trend is a shift from the domestic language to English in scientific writing (Butler 2000; Calaresu 2011). In the Nordic countries, Denmark has demonstrated an impressive growth of ophthalmologic scientific production. This is also valid for the whole field of clinical medicine, which increased by 108 per cent in 2014 compared with 1999 (NordForsk 2017). A proposed explanation is that Sweden's research underwent a relative decline compared with that of Denmark's because Swedish universities are mainly externally funded, while Danish universities have most of the resources for research at their own disposal (Öquist & Benner 2012). Centralization of resources to the capital region, increasing funds and certain productive units are other possible causes for the Danish progress (Oliver Klefter, personal communication, May 2022).

Myopia was the world's most common eye topic for research in this study, but ranked number seven in a previous study analysing topics from 2010 to 2014 (Boudry *et al*. 2016). This could probably also be interpreted as an increased impact from Asia, and especially China. In turn, this interest could be caused by an especially high prevalence of myopia in east Asia (WHO 2017).

To the best of our knowledge, no previous study of worldwide ophthalmologic research output has used such a long time frame. This is also the first study in this field comparing keywords between countries and analysing Nordic research output. This study also has several limitations. Even if the number of articles and impact points was determined for each nation, the value of individual articles was not assessed. Impact factor as an outcome measure has been criticized (Haustein & Larivière 2015), and could hide significant discrepancy in manuscript quality. However, impact factor reflects citations indirectly, as citation rates ultimately determine a journal's impact factor. Download rates of academic journals are an alternative to citation counts to measure the value of journal subscriptions. However, as these statistics are supplied only by publishers, they might not be sufficiently reliable (Wood‐Doughty *et al*. 2019).

The included journals might also not reflect the complete picture of ophthalmologic research, as these papers also could be published within other scientific fields. For example, a quarter of ophthalmic systematic reviews are published in non‐ophthalmic journals (Chen & Jhanji 2012). An additional metric not included in this study is the proportion of articles Open Access. If ophthalmologic journals with open access have higher citation metrics, which has been shown among medical journals, could therefore be analysed in further studies (AlRyalat *et al*. 2019). How the orientation of the ophthalmologic journal (reviews, translational basic science, clinical trials, *etc*.) affects the impact factor could also be a subject to additional research. It would also be possible to include more than 20 top‐ranked ophthalmological journals. However, by only including the journals with the highest impact, less significant articles might have been avoided. It should also be noted that all included journals are published in the English language, which may produce a bias towards English‐speaking countries. As Web of Science and Scopus are different datasets, combining them might also cause errors in the calculation of impact factor. At the same time, the method described might be the best available alternative and only affected the data to a minimum extent (0.9% of included articles). Due to small numbers, the trends within the Nordic countries might also be affected by minor fluctuations.

In conclusion, publications in ophthalmology have increased dramatically, with the United States as the greatest contributor but with an impressive growth in China. Among the top five ophthalmological topics, Diabetic retinopathy was the only one with a positive trend.
